# Symptomatic infections less frequent with H1N1pdm than with seasonal strains

**DOI:** 10.1371/currents.RRN1140

**Published:** 2009-12-28

**Authors:** Antoine Flahault, Xavier de Lamballerie, Thomas Hanslik, Nicolas Salez

**Affiliations:** ^*^EHESP School of Public Health; ^†^University Aix-Marseille 2 & Public Hospitals of Marseille / IRD / EHESP; ^‡^Université Versailles Saint Quentin and ^§^UMR190, Emergence des Pathologies Virales

## Abstract

A serosurvey conducted in a sample of first quarter pregnant women in France at week 48-49 of 2009 exhibit a seroprevalence level of 10.6%. It has been extrapolated in male and female population living in France mainland, aged 20-39 yr, that 1,712,000, 95%CI (1,112,700 – 2,311,300) people were recently infected by H1N1pdm (recently vaccinated women were excluded from analysis). From week 36 to 46-47 of 2009, 336,288, 95%CI (207,303-421,299) patients visited their general practitioners with clinical influenza in France, mainland. We then extrapolated the proportion of symptomatic H1N1pdm influenza in both males and females aged 20-39 yr who visited their GP to be 19.6%.

Surprisingly, 8 months after having alerted at a global level for H1N1pdm, there is still no published data on seroprevalence in any countries. Therefore, there is still no published estimation of the proportion of asymptomatic infections due to the pandemic strain. We have launched in France a weekly repeated seroprevalence survey in pregnant women who were volunteers to give their blood for H1N1pdm hemagglutination inhibition (HI) serosurvey during their first term of their pregnancy [1]. By comparing our data to cumulated figures of incident cases of clinical influenza provided by the French Sentinelles system [2] we provide estimate of the proportion of patients with symptomatic influenza presentation which led them to visiting their general practitioner.  

**Weekly repeated H1N1pdm seroprevalence survey ** We have launched a regular seroprevalence survey among a sample of consecutive pregnant women who systematically visit a laboratory from a predefined network of labs for giving their blood for toxoplasmosis serology. These women gave a written consent for providing a 50 microlitres serum sample for H1N1pdm serosurvey. Data on age and recent exposure to H1N1pdm vaccination were collected. Blood samples were then shipped to Marseilles by express mail, where a standard HI assay using human group O erythrocytes and 4 HI units of non inactivated H1N1pdm virus was performed in a L3 laboratory. Based on results of previous tests performed in a sample of the general population and on the follow up of microbiologically documented infections (manuscript in preparation), only dilution above 1/40 were considered to be relevant for detection of recent H1N1pdm infections in this age group. From serological results obtained at weeks 48-49 in pregnant women, a cumulative seroprevalence in both males and females aged 20-39 yr was estimated: 1,712,000 recent seroconversions to H1N1pdm, 95% confidence interval (1,112,700 ;  2,311,300). These estimates are published on the InVS weekly bulletin [3]. Importantly, it should be noted that, considering an observed delay of approximately 2 weeks for seroconversion,  serological results obtained at weeks 48-49 are representative of cumulated cases at week 46-47. 

## **Clinical influenza in 20-39 yr from the French Sentinel system**

The Sentinelles Network (UMR-S 707, Inserm and University Pierre and Marie Curie) is a network of 1300 volunteer general practitioners, working throughout the metropolitan regions of France (2% of the total general practitioners in these regions). This national electronic system of clinical surveillance permits the real-time surveillance of the incidence of influenza-like illness (ILI) seen in general practice. A case of ILI is defined as a sudden fever > 39°C (>102°F) with myalgia and respiratory signs. Using a periodic regression model applied to historical surveillance data, the excess consultations for influenza-like illness is estimated each week. This excess is the difference between the observed number of consultations and the expected number, under the hypothesis of no influenza circulation, estimated by the model. It can be ascribed to clinical influenza cases seen in general practice. 

Sentinelles provides an unrestricted access to its aggregated data [4].  However we had to customize our request by selecting only cases aged 20 to 39 yr and to extrapolate for all France, under assumption of representativeness of the sample of general practitioners. Cumulated extrapolations in this age group, from week 36 to week 46-47 of 2009 give the following figures: clinical cases suspected to be due to H1N1pdm, 336,288 cases,95% confidence interval  (207,303 ; 421,299).

**Calculation of the fraction of  symptomatic H1N1pdm infections who visited their general practitioners in France among the total number of infections in the age group 20-39 yr**

Basis for calculation of this fraction was the following formula:

[1 – {(SeroprevH1N1-ClinicalH1N1)/SeroprevH1N1}]

We then extrapolated the proportion of symptomatic H1N1pdm influenza in French people aged 20-39 yr who visited their GP to be 19.6%. This calculation may approach highest estimation of proportion of asymptomatic infections (i.e. 80.4%), as cases not seen by general practitioners may not only be asymptomatic cases, but also paucisymptomatic cases or symptomatic cases who did not seek any medical advice. 

*This work is funded by EHESP School of Public Health, Rennes, France.*

*Seroprevalence surveys were conducted by Unité des Virus Emergents, Université Aix-Marseille 2, faculté de Médecine de Marseille ; EHESP, Rennes, France ; Réseau de Biologie Moléculaire Libérale ; Alliance nationale pour les Sciences de la Vie et de la Santé, France ; Institut de Veille Sanitaire, Saint-Maurice, France.*

*The authors have declared that no competing interests exist.*
